# A Comparative Analysis of Explainable AI (XAI) Techniques for Transparent and Reliable Image Classification

**DOI:** 10.3390/e28050562

**Published:** 2026-05-18

**Authors:** Sovon Chakraborty, Shakib Mahmud Dipto, Kevin R. Pilkiewicz, Michael L. Mayo, Pratip Rana

**Affiliations:** 1 Department of Computer Science, Old Dominion University, Norfolk, VA 23529, USA; schak003@odu.edu (S.C.); sdipt001@odu.edu (S.M.D.); 2 Environmental Laboratory, US Army Engineer Research and Development Center, Vicksburg, MS 39180, USA; kevin.r.pilkiewicz@erdc.dren.mil (K.R.P.); michael.l.mayo@erdc.dren.mil (M.L.M.)

**Keywords:** XAI, image classification, LIME, Grad-CAM, PEEK, LRP, interpretability

## Abstract

Evaluating the trustworthiness of black-box machine learning models remains a significant methodological challenge. Their lack of transparency and interpretability limits applicability, because stakeholders often seek transparency before trusting the results of black-box machine learning models. Explainable AI (XAI) methods provide for human-understandable justifications and informed decision-making of these black-box architectures. Therefore, it is imperative to select the proper XAI model tailored to specific tasks. In this research, we focus on examining four XAI techniques: PEEK, LRP, GRAD-CAM, and LIME to understand how they perform against each other for image classification tasks. We evaluate the performance, robustness, generalizability, noise stability, and computational efficiency of these methods using a globally recognized dataset. With 7390 images, the Oxford IIT pet dataset provides a comprehensive resource for training a custom Convolutional Neural Network (CNN) and VGG16, enabling a consistent evaluation of each XAI method. First, we analyze the saliency maps of the input images and observe the regions predicted by these XAI methods, and then leverage a noise analysis approach to evaluate their performance in terms of accuracy. We further explore the robustness, run-time, and “faithfulness” metrics of each XAI method. In general, we find that these methods can identify a set of input-data features that are critical for accurate classification but also intuitive, such as the outline, face, and eyes of subjects. However, our analysis reveals only marginal consensus among XAI methods in identifying those critical features. Grad-CAM demonstrates strong robustness and stability in VGG16, but the performance on the shallow CNN model remained inconsistent.

## 1. Introduction

The swift growth of Deep Learning (DL) mandates transparency and trustworthy decision-making in domains such as computer vision and medical imaging. Interpretability, which aims to illuminate model decision-making, plays an indispensable role in enabling stakeholders to comprehend model behavior and evaluate the reliability of automated decisions. Because of the nascent nature of Explainable Artificial Intelligence (XAI) methods, identifying the most reliable, robust, and generalizable methods remains a critical challenge. Many XAI methods behave inconsistently across datasets [1], complicating their integration into real-world systems. Moreover, modern black-box models continue to exhibit increasing computational and structural complexity, raising the need for XAI techniques that can reliably interpret their decisions [2]. The primary limitation is that DL models are not often interpretable, and the decision-making process is unclear because of their black-box nature. XAI plays a pivotal role by providing explanations to interpret the decision-making process, thus ensuring transparency, accountability, and fairness [3,4].

The term “XAI method” was first introduced by the Defense Advanced Research Projects Agency (DARPA) with the aim of transforming opaque black-box systems into transparent, interpretable white-box architectures. In recent years, the role of XAI [5,6,7] has become central to trustworthy and responsible AI, where transparency, accountability, and interpretability are foundational requirements. Early XAI research classified methods into two broad categories: knowledge-driven [8] and data-driven. Symbolic or knowledge-driven XAI relies on human-understandable rules, logic, and expert reasoning, originating from early expert systems such as MYCIN [9]. However, by the 1990s, the lack of scalability and generalizability in these systems led to a shift toward data-driven machine learning (ML) approaches. To bridge the interpretability gap in black-box models, researchers subsequently blended symbolic reasoning with statistical learning, marking a significant step toward more flexible and powerful XAI techniques.

A key requirement in data-driven XAI is the ability to provide local, global, and instance-level explanations. Intrinsic models, such as Linear Regression, offer interpretability by design, whereas complex non-linear models require post hoc techniques [10]. Post hoc methods operate in either model-agnostic or model-specific ways. Local Interpretable Model-agnostic Explanations (LIME) is a widely adopted model-agnostic method, while Gradient-based Class Activation Mapping (Grad-CAM) and Layer-wise Relevance Propagation (LRP) offer class activation maps and layer-wise relevance propagation, respectively [11]. Attention-based XAI models also continue to emerge. Recent work on knowledge-based machine learning systems [12] integrates interpretability through decision trees and Bayesian networks, but inserting such structured knowledge into deep black-box architectures remains difficult. Moreover, despite achieving high performance, most black-box models lack interpretability, thus raising the importance of generating the heatmaps to explain models’ decisions [13]. The interpretation of highly parameterized nonlinear models may also suffer due to a lack of complex ground truth data [14].

Therefore, the introduction of XAI provides essential transparency and trust, yet substantial challenges persist in evaluating these methods for robustness, generalizability, and computational efficiency. The lack of consensus on the effectiveness of widely used XAI techniques further underscores the need for comparative analysis. Among existing approaches, LIME, Grad-CAM, and LRP are extensively used for image-based interpretation [15], while Probabilistic Explanations for Entropic Knowledge (PEEK) [16] has recently gained attention for its suitability in image classification tasks. However, their comparative strengths, stability under noise, and real-world feasibility remain underexplored. In this research, we focus on evaluating the performance, robustness, generalizability, noise stability, and computational efficiency of these algorithms using a globally recognized dataset.

The contributions of this research can be summarized as below:

- This study provides a detailed comparative evaluation of four widely utilized XAI methods: LIME, Grad-CAM, PEEK, and LRP in Oxford IIT pet datasets in terms of numerous performance metrics.
- Contributes to building trust in black box architectures through rigorous testing in a controlled benchmarking environment.
- Robustness of these algorithms to input noise and their generalizability to different architectures are assessed to provide a better understanding of how they can be applied to existing models.
- Our study provides a systematic benchmarking and consistency analysis framework that identifies vulnerabilities and the trade-off for each XAI method. Based on our analysis, end users can select the best XAI model for a task by understanding its advantages and limitations.

## 2. Related Work

Convolutional Neural Network (CNN)-based architectures [17,18,19] predominate image classification tasks for their strong performance in both general and medical image analysis, with a wide range of applications, including agriculture, healthcare, automotive systems, supply chains, and astronomy [20,21]. However, their deployment becomes difficult when interpretability and transparency are a concern [22], which has motivated the development of XAI methods that address these limitations by revealing internal model behavior and offering stakeholder-friendly, intuitive explanations that create confidence in model predictions. Grad-CAM [23] is a novel XAI method that generates saliency maps by projecting the backpropagated prediction gradients onto the input image. Another popular approach for CNN and related architectures is the LRP approach [24], which assigns relevance scores to each neuron back through the entire model architecture. LRP is mainly integrated with those applications wherein layerwise composition is computationally feasible. LIME [25] is an XAI approach that segments and performs patch-level predictions. Although this XAI method is computationally expensive, it creates powerful explanations that can be derived from local features, which assist in interpreting black-box architectures for clinical and security applications where high confidence in model predictions is required. PEEK is another XAI method that produces pixel-level fidelity for image classification tasks by leveraging epistemic uncertainty to quantify the diversity of feature maps. However, the broad applicability of these XAI methods remained limited due to their limitations and inconsistent behavior. For example, Gulum et al. [26] notes that medical professionals cannot accept error-prone algorithms that misclassify features in medical images. In addition, Cao et al. [27] and Loh et al. [28] note that XAI methods might perform more poorly on heterogeneous input data that combine images and biometric data with clinical notes. Most hospital systems encounter challenges because of suboptimal visualization quality and non-intuitive XAI interfaces [29], where another study [30] reflects that post hoc methods like LIME can provide incomplete explanations.

Our motivating hypothesis is that if these methods aim to identify critical elements of input data used by the CNN for accurate image classification, then the image subsets (groups of pixels) identified by these methods should be highly correlated. To test this hypothesis, we explore four XAI techniques, namely LIME, LRP, Grad-CAM, and PEEK, and evaluate their performance across a variety of metrics. These methods provide diverse explanation strategies, including perturbation-based and gradient-based approaches. We test for their classification robustness and stability, and runtime and also evaluate “faithfulness” using a pixel-perturbation approach. The literature shows that most studies are focusing on applying XAI techniques for research tasks, including computer vision. In [31], authors effectively used SHAP and LIME to improve the interpretability of the complex models. The aim was to improve transparency. They compared the results of both methods to build trust in predictive maintenance systems. The limitation relies on the rigorous evaluation of the XAI methods, where it is merely utilized as a supporting tool rather than being analyzed deeply. Gupta et al. [32] analyzed four XAI methods, namely LIME, SHAP, PDP, and Decision Tree, in the healthcare domain. Moreover, the global and local interpretability of these techniques is discussed, but the performance of the XAI methods under different situations is not explored. Comprehensive benchmarking regarding the evaluation of XAI methods has been performed in [33,34], where authors have considered faithfulness, stability, and robustness for evaluation. The evaluation takes only tabular data, where they have not considered any image dataset for evaluation purposes. Another study [35] considered image datasets but did not employ any proper evaluation technique other than faithfulness.

Here, after the critical evaluation from the literature, we aim to identify key advantages and limitations of these four XAI methods under controlled conditions. The goal is to evaluate the XAI methods under controlled settings with respect to image datasets that will assist the researcher in this domain in identifying suitable XAI methods for specific tasks.

## 3. Methodology

**Figure 1:**
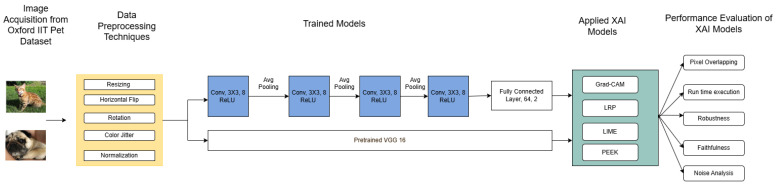
Overview of the methodological pipeline used in this study. The workflow consists of three main stages: (1) image preprocessing using resizing, augmentation, and normalization; (2) model training with a custom CNN and pretrained VGG16; and (3) application of XAI methods (Grad-CAM, LRP, PEEK, and LIME) followed by performance evaluation using overlapping region in saliency maps, runtime, robustness, faithfulness, and noise analysis.

This section describes our implementation and performance-measuring setup, and Figure 1 illustrates our workflow. We first gathered data from the Oxford IIT Pet dataset [36], which consists of cat and dog images that are preprocessed and provided to a shallow custom CNN and a pre-trained VGG16 architecture (which is further finetuned). All four XAI methods were applied to this model. XAI algorithm performance is examined by quantifying the extent of the overlapping region in the saliency maps, an application of noise analysis, and an assessment of robustness, faithfulness, and run-time execution. These steps are explained in more detail below.

### 3.1. Dataset Description

To compare these XAI methods, we used the Oxford-IIIT Pet dataset, which is specialized for training tasks in computer vision, especially image classification, object detection, and semantic segmentation. The Visual Geometry Group (VGG) released this dataset by capturing different breeds of cats and dogs. The images are annotated as cat or dog, and a total of 7390 high-quality images are stored in RGB format. Of these images, 1478, which is 20% of the whole image dataset, are identified as the test dataset for our comparative analysis. We selected this dataset for its intra-class variability and suitability for image classification tasks, making it well aligned with DL architectures.

### 3.2. Preprocessing of Dataset

The images from the dataset undergo a series of pre-processing steps prior to model training to remove unwanted artifacts and so that critical features can be extracted efficiently. After this data preparation phase, the model will converge proficiently and produce stable, high-quality representations during training while reducing the potentially redundant and irrelevant background. Table 1 shows the preprocessing techniques used here. Images in this dataset varied in size, so we leveraged Python’s 3.14.4 ‘resize’ function to convert all images into a more uniform 224 × 224 pixel format. For the improvement of generalizability and robustness, the data augmentation techniques RandomHorizontalFlip() and RandomRotation of 10 degrees have been applied in the training phase. Furthermore, in the case of random changes of brightness, contrast, saturation, and hue, the Colorjitter function from PyTorch 2.2.0 is applied. All the values are set to 0.1 for improved generalization purposes. Furthermore, the ToTensor() method converts a PIL image into a pytorch tensor by changing its shape and rescaling the pixel values. To maintain consistency among the models and improve performance, normalization is performed based on mean and standard deviation.

### 3.3. Test Models: Role of CNN Architecture Complexity

We select the popular VGG16 model, in addition to a custom “shallow” CNN, to understand the relative performance of each of the four XAI methods tested here. These models will help us to assess the performance of XAI methodologies in both simple and complex network architectures.

The custom CNN architecture is constructed with a feature extraction module, which has four sequential convolutional blocks. The purpose of each block is to progressively learn more abstract image representation. In the very first layer, 3 input channels and 8 output feature maps are applied using a 3 × 3 kernel. The stride and padding are set to 1, which preserves the spatial resolution of the input image. A ReLU activation is placed after that, with in-place computation enabled, allowing efficient memory usage while introducing non-linearity into the network. The spatial dimension is reduced to half after a subsequent Avgpool2D layer with a 2 × 2 kernel and a stride of 2. The pattern of the first convolution block is repeated in the second block, where 8 input channels and 16 output channels with a 3 × 3 kernel, a stride of 1, and a padding of 1 are provided. The third convolutional block expands the representational capacity by applying 16 input channels and 32 output channels. The kernel, stride, activation function, and padding size remain identical, allowing the model to focus on abstract structures such as shapes and object parts. Finally, in the fourth convolutional block, the channel is further expanded from 32 to 64. The average pooling layer with a kernel size of 3 and a stride value of 2 compresses the feature representation into compact 64 × 14 × 14 feature maps. Across all four blocks, the multistage pooling reduces the dimensions significantly.

VGG16 is a state-of-the-art architecture in such cases, where it applies 3 × 3 filters throughout the network that capture proper features. Similarly, like the custom CNN, a ReLU activation function and max pooling layer are followed to reduce spatial dimensions. The complex hierarchical features are learned for high-level tasks. The fully connected layers of VGG16 perform as dense classifiers that convert the extracted features into final classification. Another reason for performing better is pretraining on the ImageNet class.

Table 2 illustrates hyperparameter tuning. The batch size is set to 32 for both architectures, whereas the number of epochs is 300 for the custom CNN model but 5 for VGG16. As a pretrained model on ImageNet, VGG16 can understand the complex features much easier than the custom CNN. CrossEntropyLoss is used for the calculation of the loss function for both cases, and the Adam optimizer is utilized. The learning rate is set to $1\times 10^{-4}$, and only 2 classes are classified as cats and dogs in the final classification.

### 3.4. Methods and Models for Explainable AI (XAI)

In terms of image processing, we consider four XAI methods: LIME, PEEK, LRP, and Grad-CAM. We refer to any model as an “XAI method,” if it takes the model parameters or weights as inputs and processes them to understand the overlapping region in the saliency maps of the input image.

The cardinal purpose of selecting LIME lies in its post hoc interpretability, which aims to explain the prediction of a black-box model by approximating it locally with an interpretable surrogate model. LIME perturbs the input data and the corresponding outputs, from which it builds a simplified linear model of the process, and most contributing features remain understood by the linear model. The model-agnostic behavior of LIME makes it suitable to be applied in numerous deep learning or machine learning algorithms. However, LIME suffers from instability due to its random sampling nature and is limited to local interpretability only.

Grad-CAM is a gradient-based method that generates localization maps that highlight influential regions of input data that reportedly contribute to class predictions made by an arbitrarily complex, “black-box” CNN model. Grad-CAM computes the gradient of the predicted class in relation to feature maps. In this way, it identifies subsets of the input data presumably correlated with a model’s decisions.

In contrast with Grad-CAM, the LRP method falls into the gradient-free category and is tailored specifically for deep neural networks. The outputs of a CNN model are composed of propagating relevance scores backward through the convolutional layers. A score is calculated during this procedure, in which each neuron redistributes its relevance to the neurons in the previous layer proportionally to their contribution. LRP is designed for tasks related to medical imaging and computer vision but requires internal access to the model’s architecture and parameters.

PEEK is a statistical approach that analyzes the feature maps (also called latent representations) within convolution layers, which are stored as 3D tensors: $L\in R^{l\times w\times d}$, wherein *l* and *w* are, respectively, the height and width of the feature maps, and *d* is the number of channels. Each channel captures different aspects of the image, such as edges, textures, or color patterns, which the model uses to understand and recognize objects in the input. PEEK is interesting because it essentially identifies subsets of input data associated with a greater diversity of convolutional transformations. In other words, it identifies input data for which the model leverages a greater number of resources to assess. One possible interpretation for this behavior is that more convolutional transforms are required to evaluate more informationally dense input data. The PEEK approach hypothesizes that input data with high information density should be more highly correlated with the class label.

### 3.5. Performance Metrics and Criteria

#### 3.5.1. Jaccard Score: Quantifying Pixel Similarity

For image processing and computer vision tasks, the overlapping of regions of the saliency map provides a broader view when images are processed in segments or patches. Here, “overlapping” denotes the common highlighted regions in the saliency maps generated by two XAI methods. In this research, the Jaccard score is being used to understand the overlap of regions across four different methods. The Jaccard score, *J*, can help to measure the similarity between two sets of pixels, which measures the similarity of two sets *A* and *B* in terms of their “overlap.” Formally, it can be calculated as the ratio of the size of their intersection, |A∩B|, to the size of their union, |A∪B|:

(1) $$J=\frac{\left|A\cap B\right|}{\left|A\cup B\right|},$$

In image segmentation tasks, the Jaccard score measures the overlap between the predicted segmentation mask and the ground truth mask, wherein a unit score indicates perfect overlap and 0 indicates no overlap (i.e., an empty intersection). This method of pixel similarity offers a reliable and quantitative means to compare XAI methodologies.

#### 3.5.2. Run-Time for Assessment of XAI Complexity

The performance of each XAI method can be assessed in terms of run-time execution. Run-time is a significant consideration in decision-making across industries such as healthcare and finance and in situations where automated systems must react quickly, for example, to predict faults or act to prevent failure. In principle, run-time is correlated with the number of operations needed to execute a model, so run-time may also serve as a pragmatic measure of model complexity. Thus, we assess run-time for each XAI method using two completely different architectures: the more complicated VGG16 and our comparatively much simpler custom CNN model.

#### 3.5.3. Robustness of XAI Predictions to Model Perturbation

How dependent are the predictions of XAI methods on the CNN model? We can address this question by assessing the predictions from each XAI method for models that differ only by small changes to their pretrained weights. Laplacian noise is utilized here to understand the sensitivity in the predictions of XAI methods. As a model is perturbed further from its trained “optimum” via slight exploration of the volume of parameter space associated with the weight values, we should expect its overall performance to decrease and the XAI methods to degrade. The manner in which the input data subsets change may reveal which identified pixels are useful for classification and which pixels could be chosen in error by the XAI method.

#### 3.5.4. Faithfulness

For measuring the performance of XAI models, faithfulness is vital, as it describes how well an explanation truly reflects what the model actually uses to make predictions. Incorrect interpretation can be generated from an unfaithful explanation, especially in image analysis. To validate the reliability and credibility of an XAI model, assessing faithfulness is vital. We consider an explanation as “faithful” if the identified image regions have a genuine impact on the model’s output. To measure faithfulness, we set the intensity of the most important 10% of image pixels to zero. If model performance decreases, then we conclude the XAI method successfully identifies pixels that are important for image classification and, therefore, can be considered more “faithful”.

#### 3.5.5. The Effect of Input Noise

One of our targets is to assess the models’ robustness and reliability in terms of “noise.” Pixels are spatially distinct elements of color, which raises a question of whether the degree of color intensity is correlated with model performance. To assess this, we will resample the colors of certain pixels from an input image, chosen at random from some distribution decided later. Once the pixel elements are altered, two metrics are calculated, including the percentage of correct predictions and the probability that the true class is identified. A model’s accuracy under increasing levels of noise (larger variance) is observed in the first case, whereas the second criterion observes that the confidence in the true class degrades with noise.

#### 3.5.6. Theoretical Grounding of Evaluation Metrics

We have used several metrics in our study, such as Jaccard similarity, robustness, and faithfulness. These metrics are evaluated empirically; they are deep-rooted in information theory and uncertainty quantification [37]. In information-theoretic terms, robustness indicates high information consistency between a clear explanation and a noisy explanation. Faithfulness assesses whether masking features degrade model performance. Theoretically, making an informative feature should maximize the predictive uncertainty. Furthermore, PEEK is grounded in information-theoretic principles. Specifically, PEEK calculates the Shannon entropy associated with all feature maps of a convolutional layer. One hypothesis is that convolutional neural networks solve image-classification tasks by assigning more feature maps during training to discriminate the informationally dense regions of an image that are correlated with accurate classification, such as the face, body, or outline of a subject. Thus, comparison of XAI methods against PEEK results can help to contextualize their relationship with the informational character of an image.

## 4. Results and Discussion

**Figure 2:**
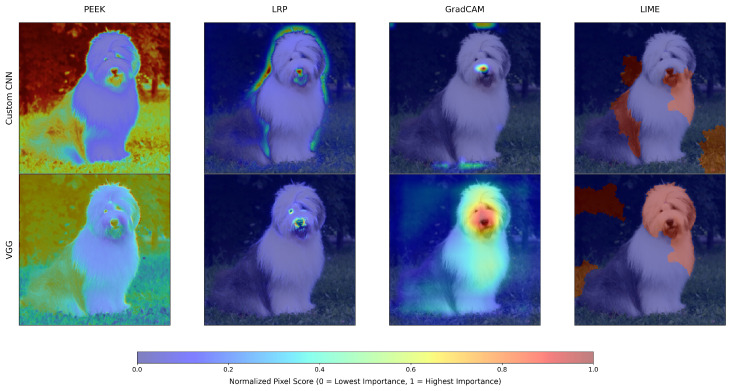
Comparative visualization of explainability outputs, illustrating how four XAI techniques generate distinct saliency maps for the custom CNN and VGG16 models. The heatmaps are normalized using min-max normalization.

We have run the four XAI methods on the test data. The output saliency maps of XAI methods for a single image, using both the CNN and VGG16 models, are shown in Figure 2. Here, the saliency map is normalized between 0 and 1 to improve the visualization. This image shows that each XAI method emphasizes different regions of the saliency map. In this image, PEEK focuses on the body and the background. LRP focuses on most of the facial region and the object’s boundary. Grad-CAM mainly selects the facial part, particularly for the VGG model. LIME selects the object’s face and body, as well as the background.

### 4.1. Regions Overlapping Reveals a Marginal Consensus Across XAI Methods

We start to understand the consensus among XAI methods on the test data. Our first aim is to understand the region overlapped in XAI methods. Here, pixel overlapping denotes whether the XAI methods highlight the same pixels when identifying a cat or a dog in the saliency maps. One of the important globally utilized metrics for understanding pixel overlapping is the Jaccard score [38] that usually ranges from 0 to 1, where 0 denotes no overlapping of pixels between two XAI models. On the contrary, a high Jaccard score defines a high overlap of pixels. We considered only the top 10% pixels from the saliency maps for computing the Jaccard score as we focus on the important regions that influenced the decision. Past studies suggest that consideration of more pixels may not be beneficial [39]. Considering more pixels might highlight irrelevant background regions that have the probability to add noise in evaluation. Therefore, we are restricting our selection to only the top 10% pixels.

**Figure 3:**
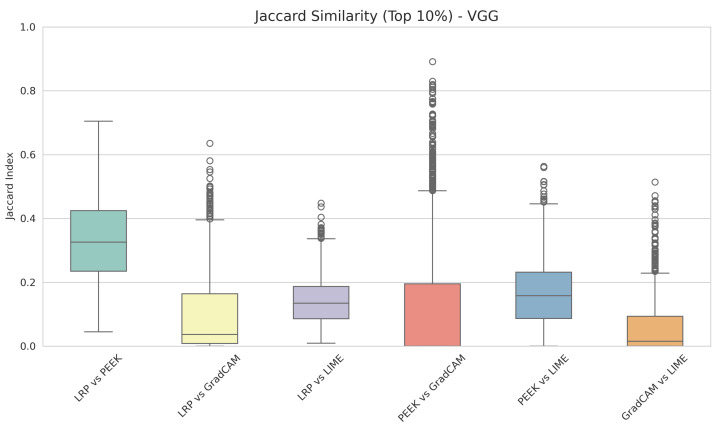
Box plot of the Jaccard index across XAI method pairs for the custom VGG16 model.

Figure 3 depicts the box plot of the Jaccard index for all the XAI method pairs for the VGG16 architecture. The illustration provides the consensus among XAI methods. The maximum overlapping of pixels is achieved by LRP and PEEK. The median of the Jaccard index of these two XAI methods is 0.32. On the contrary, Grad-CAM and LRP have the lowest median score of 0.13; thus, both XAI methods capture a different set of important pixels for the model’s prediction. Similar patterns are also observed in the PEEK and LIME consensus. So, from this, we can declare that XAI methods are reflecting a minimum consensus among them while highlighting important regions.

**Figure 4:**
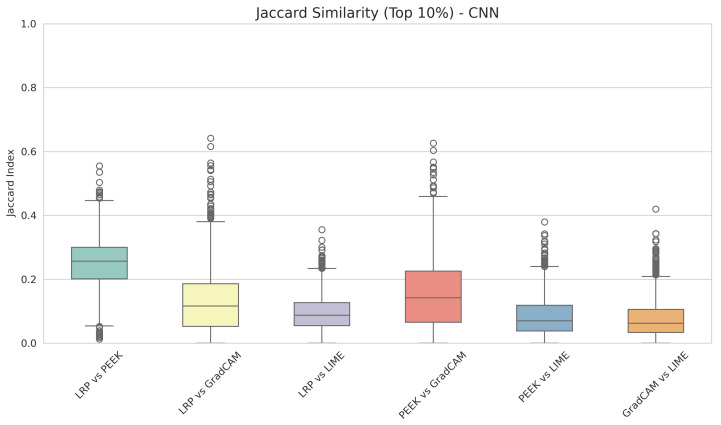
Box plot of the Jaccard index across XAI method pairs for the custom CNN model.

Next, we focus on the overlapping of pixels in our custom CNN model. Consistent with the VGG16 model, Figure 4 exhibits that the maximum overlapping of pixels is observed in LRP and PEEK, i.e., almost 0.27. Grad-CAM and LIME, along with PEEK and LIME, have the lowest similarity, which is nearly 0.14. Finally, PEEK and Grad-CAM show a median of 0.17, which is not superior to other pairs. These results are relatively similar to those of the VGG; however, in most cases, we observed lower overlap with the CNN model than with the VGG16 model. This suggests that when the model is well-optimized, the agreement of the XAI methods generally improves. For well optimized models, XAI methods extract more stable and detailed features from the layers that eventually produce more consistent saliency maps.

### 4.2. PEEK Exhibits the Lowest Average Runtime for Both Lightweight CNN and VGG16 Architectures

Our analysis of pixel overlapping shows that the different XAI methods have captured different pixel sets for explaining the image. That does not directly express the performance of XAI methods but rather shows the agreement among them in highlighting the saliency map. Next, we aim to observe the runtime execution of the XAI methods, as it gives a broader look at how quickly an XAI method can generate its explanation.

**Figure 5:**
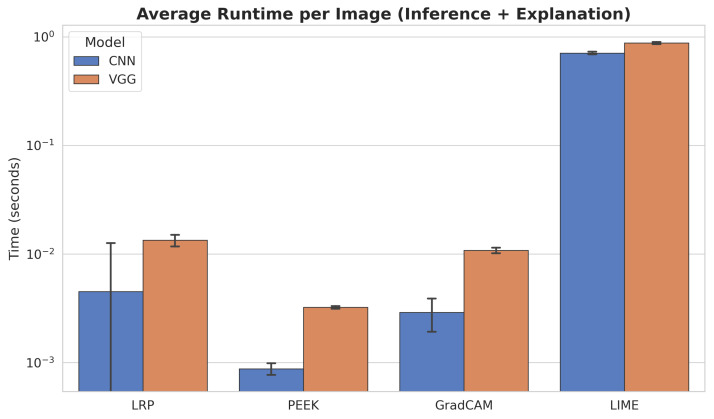
Average runtime per image for inference and explanation across XAI methods. PEEK and LRP show the lowest execution times for both CNN and VGG16 models. Grad-CAM requires more time, especially in the VGG16 pipeline. LIME is the slowest by a large margin, dominating overall runtime for both architectures.

Figure 5 provides a detailed overview of the evaluation of the runtime behavior (inference + explanation). To grasp the full understanding, we have taken 300 iterations into account, and the values are reported in a logarithmic scale. To reflect the performance variability, we incorporated the standard deviation of the runtime. PEEK is the fastest XAI method in both the custom CNN and VGG16 architectures. In the case of the shallow CNN architecture, PEEK achieves the result in less than 0.001 seconds with a low standard deviation. Likewise, in VGG, the runtime for PEEK is less than 0.01 s.

Conversely, LIME is the slowest method, with a runtime for both VGG16 and a custom CNN approaching 1 s. The architecture of LIME indicates that it needs to generate a large number of perturbed samples for each input. Moreover, the evaluation of these samples is placed to fit into a local surrogate model. This model-agnostic approach takes place repeatedly and independently for every instance, making the computation inherently iterative. The standard deviation for LIME is 0.02 and 0.015 consecutively for the custom CNN and VGG. After PEEK, the second swiftest method is Grad-CAM, where the approximate runtime is 0.01 in VGG16 and 0.005 for the custom CNN. Finally, LRP demonstrates faster execution than LIME, although it remains less efficient than PEEK and Grad-CAM.

Across all methods, we conclude that VGG16 model consistently requires more computation time than CNN model. The analysis reveals that model depth significantly influences explanation latency. Deeper architectures amplify computational costs, suggesting that PEEK and LRP may be more appropriate for real-time applications due to their speed advantages, while LIME offers deep insights, it suffers from high latency.

### 4.3. Grad-CAM Demonstrates Strong Robustness in VGG16, with LIME Showing Some Robustness in the Shallow CNN

**Figure 6:**
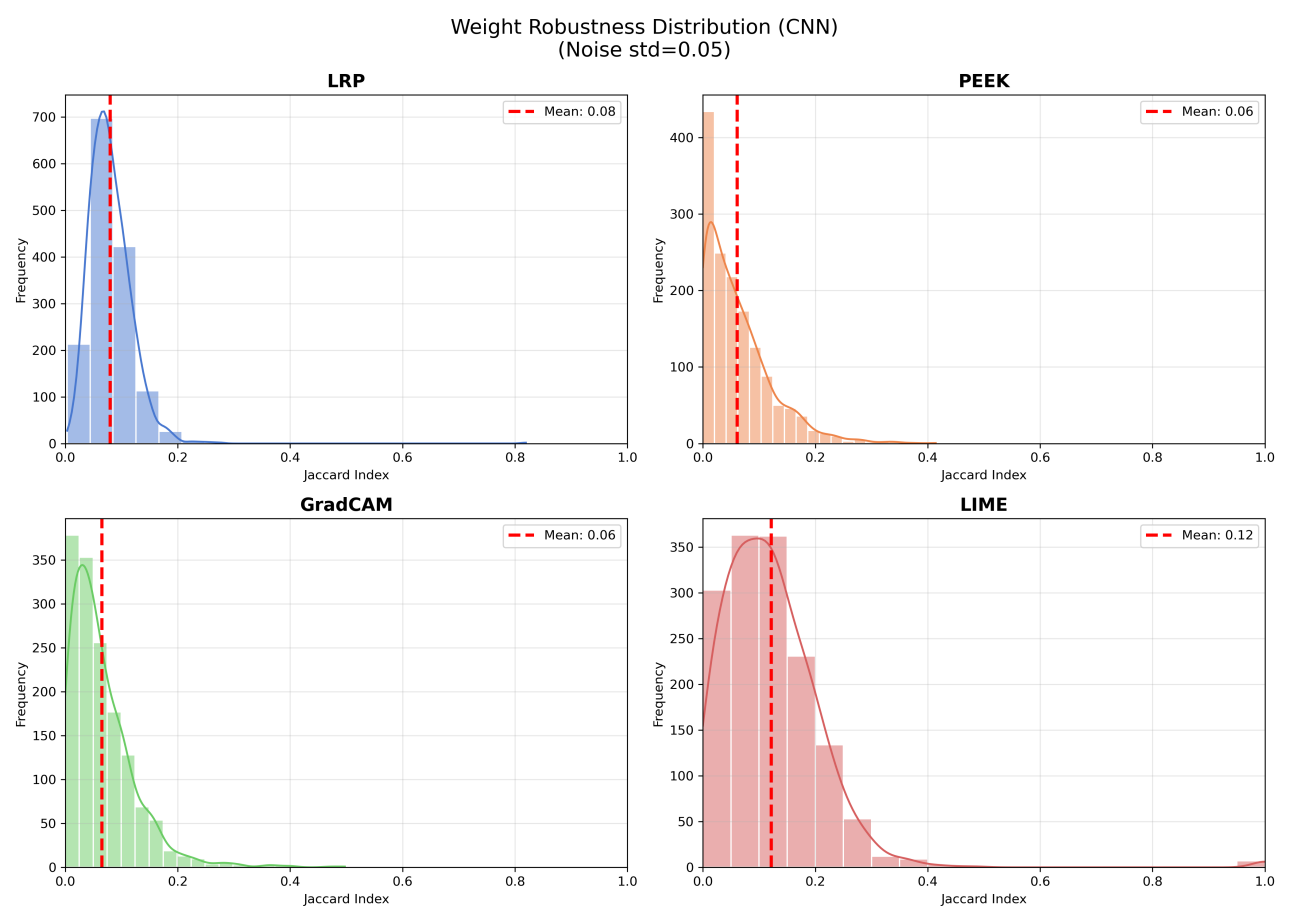
Jaccard similarity between saliency maps for our CNN model with clean and Laplacian-noised weights across the chosen XAI methods. LIME shows the highest robustness with a mean of 0.12, whereas Grad-CAM exhibits the strongest degradation; LRP remains moderately stable (mean ≈ 0.08), and LIME shows the highest stability with a mean of 0.21.

Robustness of the saliency map reflects how a small change in the values of the model’s weight parameters affects its behavior. For this purpose, the top 10% pixels of the saliency maps were used. The Jaccard index is computed by comparing the top saliency map pixels after running the xAI method with original and perturbed weights. Figure 6 depicts the robustness of the explanation methods when an additive Gaussian noise (σ=0.05) is added to the trained weights of CNN and VGG16 models. For the CNN architecture, the distribution of the Jaccard index is concentrated below 0.2. The overlap of the saliency map is minimal in most cases, with the mean Jaccard index ranging from 0.06 to 0.12. LRP shows a mean Jaccard score of 0.08, with a moderate spread. Most values range from 0.05 to 0.15, with a small number of outliers. PEEK shows the least robustness score in the CNN model, with a mean of only 0.06, indicating that the output frequently fluctuates under weight perturbation. Grad-CAM also exhibited a similar mean Jaccard score to PEEK, suggesting that in the CNN model, small changes in the weights cause sensitive fluctuations. Among the four XAI methods, LIME yields a mean of 0.12 on the shallow CNN model. The distribution is broad, with observed samples clustered around 0.1 to 0.3. In the shallow CNN, LIME has the most stability among other XAI methods.

**Figure 7:**
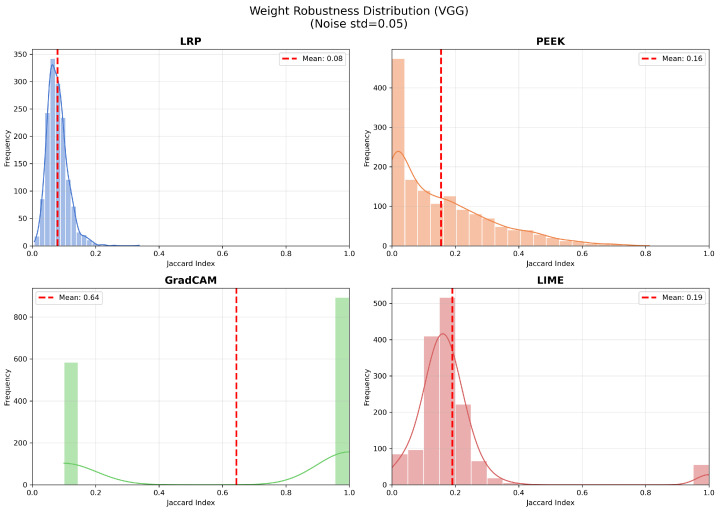
Jaccard similarity between saliency maps for VGG16 with clean and Laplacian-noised weights across the chosen XAI methods. LRP shows the lowest robustness with scores near zero (mean ≈0.08), PEEK and LIME exhibit moderate stability (means 0.16 and 0.19), while Grad-CAM achieves the highest robustness with a mean of 0.64.

For the VGG16, the scenario is different in the case of Grad-CAM. From Figure 7, we see that Grad-CAM has the highest mean of 0.64, which indicates strong performance in the deeper architectures. The pattern is similar to CNN for LRP, where the standard deviation is found to be 0.03 for the mean. The performance of PEEK is also improved in the VGG16 with a mean of 0.16, and the distribution is also broader. The mean of LIME is also increased by a slight margin compared to CNN, which represents similar well-trained models in both VGG16 and shallow CNN architecture.

### 4.4. Grad-CAM Depicts Most Faithfulness in VGG16 Where PEEK Underperforms in Both

**Figure 8:**
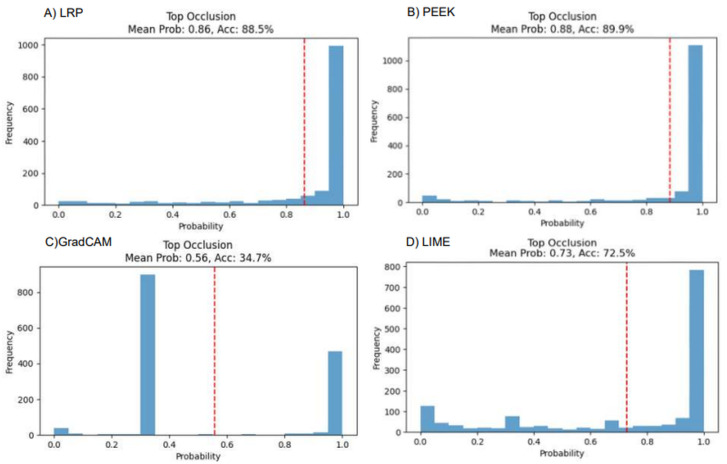
Performance analysis of four XAI models using occlusion-based evaluation, illustrating how masking different image regions affects model confidence.

The faithfulness of each XAI method for both VGG16 and CNN models can be assessed by passing an image to the model and setting the intensity of the top 10% pixels (based on saliency map) to zero. The expectation is that, after setting the pixels, the result will degrade as the most important pixels are being set to zero. From Figure 8, it shows that Grad-CAM has the steepest decline in the performance. The mean true class probability has become 0.56, and the accuracy is found to be 34.7%. In other cases, PEEK has the highest accuracy with 89.9% and LRP has the second highest mean probability with 0.86. The performance of LIME is also dropped to 72.5%, which indicates moderate faithfulness in the VGG16 architecture.

**Figure 9:**
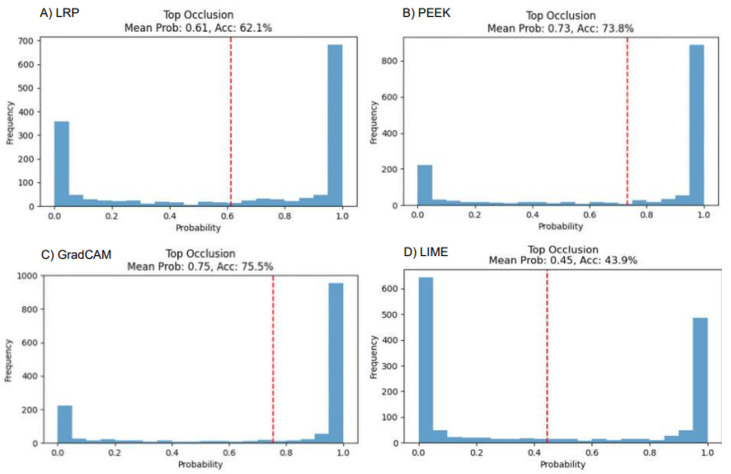
Performance analysis of four XAI models using occlusion-based evaluation in a custom CNN.

We now consider the custom CNN architecture, wherein LRP has shown the most “faithful” behavior in the case of the CNN model. The mean probability is 0.61, and accuracy is dropped to 62.1%. Grad-CAM shows a different behavior than VGG16, where the accuracy is 75.7% and the mean probability is 0.75. LIME has demonstrated only 43.9% accuracy, showing the most faithful behavior in the custom CNN model (Figure 9).

### 4.5. Model Performance Degrades When the XAI Methods’ Selected Points Are Noised

To evaluate whether XAI methods select relevant points for classification, we performed a novel noise-based analysis. First, we ran XAI methods on the test images to get the saliency map. Next, we added Gaussian noise to the selected pixels of the original image based on the map’s relevance score. Pixels containing the top 10% relevance score are called “top” pixels, the least important pixels are called “bottom” pixels, and when pixels are selected randomly, they are called “random” pixels. Gaussian noise of various magnitudes is added to “Top”, “Bottom”, and “Random” pixels of the original image, and model performance is measured.

**Figure 10:**
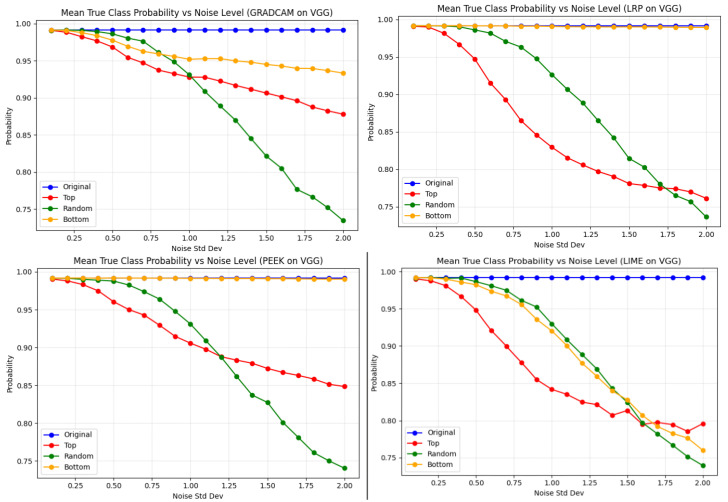
Effect of noise on true-class probability for the VGG16 model across regions of input data identified by the various XAI methodologies. Random-pixel perturbations cause the largest drop in probability across all methods at high noise levels, while bottom-region noise has the weakest effect. Top-pixel noise leads to rapid initial degradation; however, it stabilizes near 0.85–0.90 at higher noise levels. Grad-CAM shows the slowest decline and highest resilience, whereas LRP and LIME degrade more sharply, especially under random pixel noise.

Figure 10 illustrates how model performance decreases if we reduce the information encoded by the top 10% of pixels identified by each XAI method. This is achieved by adding noise directly to the original pixel values, where the additive noise is sampled from a zero-mean Gaussian distribution with standard deviation σ∈[0,2]. At first, we observed the performance of the XAI methods in the VGG16 architecture. The expectation is that with increasing value of Gaussian noise in the top pixels, the performance should degrade. The red line in Figure 10 shows that, the performance of LRP degraded the most when the top pixels are injected with Gaussian noise. On the other hand, Grad-CAM is less affected among four XAI methods in the case of top-pixel noise. Moreover, in this particular case Grad-CAM has the lowest degradation even after being noised with top pixels. For the bottom pixels, LRP, and PEEK show a stable performance, whereas LIME has a drastic fall in performance. As LIME relies more on perturbations to construct a local surrogate model, it is more sensitive to noise for any pixel, including bottom pixels also. Inversely, gradient and relevance-based methods do not suffer in such cases, as they do not rely on perturbations of the input.

**Figure 11:**
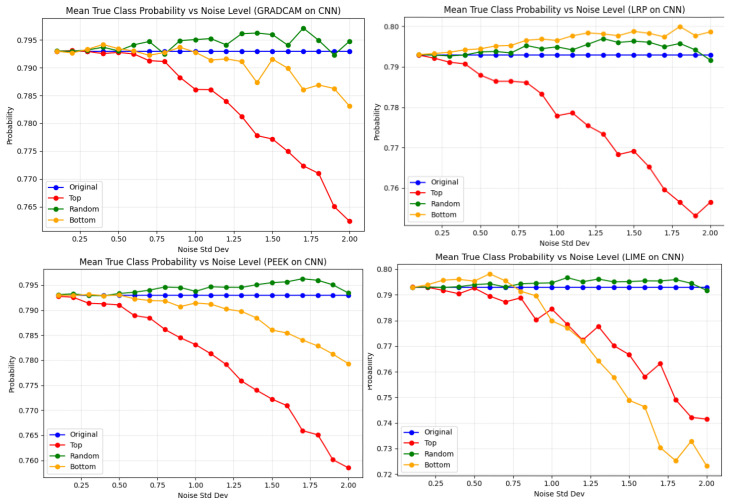
Effect of noise on true-class probability for the CNN model across XAI-guided regions. Top-region sensitivity: All methods show the steepest decline under top-region noise, confirming this area contains the most discriminative features for CNN classification, especially for LRP. Random and bottom regions: Random-region noise produces only mild degradation, while bottom-region noise shows the highest stability, with most methods staying above 0.785–0.790. Method comparison: LRP remains relatively robust, whereas PEEK shows the strongest instability.

Figure 11 provides a detailed overview of the mean true-class probability of the CNN model, which changes with increasing Gaussian noise. In the case of Grad-CAM, the pattern of performance degradation is similar to that of VGG16, except for the top pixel and the random pixel. Previously, in VGG16, random noise caused the largest performance drop, whereas in the CNN model, performance is most degraded when noise is injected into the top-scoring pixels. A similar trend is also observed in the case of the LRP XAI method for top-pixel noise. On the other hand, noise injected into other pixels does not affect the performance. For Grad-Cam, the mean true class probability falls to 0.765, whereas for LRP it was 0.755. Discriminating between these two XAI methods, the bottom-pixel noise affects the performance in Grad-CAM a little but not for LRP. Interestingly, a different trend is exhibited by LIME, where the bottom-pixel noise affects the performance of the model even more than top-pixel noise. The application of surrogate models for perturbation is the primary cause for such behavior in this particular XAI method. As expected, the least vulnerability is observed in the noise applied to the bottom pixels for Grad-CAM, LRP, and PEEK, with probability values around 0.790 for the first two and 0.782 for PEEK.

## 5. Conclusions and Future Work

In this work, we evaluate the performance, robustness, noise stability, and computational efficiency of four XAI methods for the image classification task. With careful evaluation, this study identifies the advantages and limitations of each XAI method and the scenarios in which they succeed or struggle. For example, Grad-CAM might be a better choice for the deep models rather than a shallow CNN architecture. In any case where runtime is a key requirement, PEEK can be utilized. In terms of pixel overlapping, LRP and PEEK have the zenith mean, which means that these methods highlight more consistent and focused regions of interest compared to the others. All the XAI methods demonstrate limited agreement in terms of the degree of overlap among their top-scoring pixels. The lowest similarity among top-scoring sets of pixels is found between Grad-CAM and LRP, and between Grad-CAM and LIME, consecutively, in VGG16 and a CNN model.

In runtime execution, PEEK demonstrates computational efficiency across both experimental architectures. Noise analysis shows an interesting result when both architectures are affected when the LRP-selected points are noised in the top region. The surrogate model’s application in LIME exhibits a unique pattern for bottom noise. Regarding robustness, Grad-CAM achieves the strongest performance, whereas LRP is the least robust for the VGG16 architecture.

Regarding faithfulness, a similar pattern is also observed in PEEK, where it is the least faithful model. Moreover, Grad-CAM achieves excellent performance on VGG16, indicating that this method performs well when the model is well-trained. Grad-CAM exhibits stronger performance in the deeper architecture because of its reliance on high-level feature maps. These feature maps capture more stable and semantically meaningful information.

Sensitivity to noise and local variations decreases as gradients are aggregated in deeper layers. On the contrary, LRP can redistribute relevance across all layers, leading to more spatially distributed attributions. Discussing PEEK, it performs faster because it understands pixel importance with a limited number of sampled perturbations. That is why PEEK has the lowest runtime among other models. Finally, LIME’s instability arises from its stochastic perturbation sampling and local surrogate fitting. This introduces variance across runs and reduces consistency in the presence of noise. This mechanistic difference explains our experimental observations, specifically why Grad-CAM achieves higher robustness scores in VGG16 and LRP exhibits higher overlap metrics, while LIME demonstrates higher variance.

It would be interesting to assess whether these XAI methods generalize to all image-based datasets, particularly in areas such as medical informatics, to better understand how AI models make decisions from medical images. In terms of shortcomings, we have applied only Gaussian noise, which can be expanded by introducing other noise types and adversarial perturbations. The efficacy of this research is to increase the transparency, trust, and accountability in modern AI systems. Understanding the strengths and limitations is vital, as these architectures are progressively shifting towards real-life systems. This research introduces a different perspective that will enable safer, more interpretable decision-making. With the results, the researchers will be able to select the most appropriate XAI method for their use cases, balancing performance and computational constraints.

## Figures and Tables

**Table 1:** Image preprocessing steps applied before training.

Step	Transformation
1	Resize to (224, 224)
2	RandomHorizontalFlip()
3	RandomRotation(10)
4	ColorJitter(0.1, 0.1, 0.1, 0.1)
5	ToTensor()
6	Normalize([0.485, 0.456, 0.406], [0.229, 0.224, 0.225])

**Table 2:** Training hyperparameters used in the model.

Hyperparameter	Value
Batch size	32
Shuffle	True
Learning rate	$1\times 10^{-4}$
Optimizer	Adam
Loss function	CrossEntropyLoss
Number of epochs	300 (CNN), 5 (VGG1616)
Hidden layer size	64
Output classes	2

## Data Availability

No new data were created or analyzed in this study. All the codes and data are available in the mentioned Github link: https://github.com/AMLRL-ODU/CompareXAI (accessed on 11 February 2026).
